# The Prognostic Role of RDW in Hospitalized Heart Failure Patients with and Without Chronic Kidney Disease

**DOI:** 10.3390/jcm13237395

**Published:** 2024-12-04

**Authors:** Grigorios Giamouzis, Christos Kourek, Dimitrios E. Magouliotis, Alexandros Briasoulis, George E. Zakynthinos, Assaf Sawafta, Nikolaos Iakovis, Georgios Afxonidis, Kyriakos Spiliopoulos, Filippos Triposkiadis, Thanos Athanasiou, John Skoularigis, Andrew Xanthopoulos

**Affiliations:** 1 Department of Cardiology, University Hospital of Larissa, 41334 Larissa, Greece; 2 Department of Cardiology, 417 Army Share Fund Hospital of Athens (NIMTS), 11521 Athens, Greece; 3 Department of Cardiac Surgery Research, Lankenau Institute for Medical Research, Main Line Health, Wynnewood, PA 19096, USA; dimitrios.magouliotis.18@alumni.ucl.ac.uk; 4 Medical School of Athens, National and Kapodistrian University of Athens, 15772 Athens, Greece; 5 Critical Care Department, University Hospital of Larissa, Faculty of Medicine, University of Thessaly, 41110 Larissa, Greece; gzakynthinos2@gmail.com; 6 Cardiothoracic Surgery Department, General University Hospital of Larissa, University of Thessaly, 41110 Larissa, Greece; afxonidis.g@gmail.com (G.A.); spiliopoulos@med.uth.gr (K.S.); 7 Department of Surgery and Cancer, Imperial College London, London SW7 2AZ, UK; t.athanasiou@imperial.ac.uk

**Keywords:** red blood cell distribution, width, chronic kidney disease, prognosis, mortality, heart failure, renin–angiotensin–aldosterone system

## Abstract

**Background:** Chronic kidney disease (CKD) and heart failure (HF) are interrelated conditions that exacerbate each other through mechanisms like fluid retention, neurohormonal activation, and inflammation. Red blood cell distribution width (RDW), a measure of red blood cell size variability, has emerged as a potential prognostic marker in HF. This study aimed to assess the prognostic value of RDW in HF patients, both with and without CKD, focusing on all-cause mortality and HF rehospitalizations. **Methods:** This observational retrospective study included 171 patients hospitalized for acute decompensated HF in a tertiary university hospital in Greece. Patients were divided into two groups based on their estimated glomerular filtration rate (eGFR), as Group 1 (eGFR < 60 mL/min/1.73 m^2^) and Group 2 (eGFR ≥ 60 mL/min/1.73 m^2^). RDW was measured upon admission, and outcomes of interest were all-cause mortality and HF rehospitalizations over a median follow-up period of 6.1 months. Statistical analyses included Kaplan–Meier survival curves, whereas the discrimination traits of RDW were evaluated by constructing receiver operating characteristic (ROC) curves and by calculating the area under the ROC curve (AUC). A *p*-value <0.05 was indicative of a statistically important result. **Results:** Patients in Group 1 (eGFR < 60 mL/min/1.73 m^2^) were older (80 (73–86) vs. 75 (62–83)) and manifested higher median RDW values (16.6 (15.0–18.8) vs. 15.6 (14.1–17.8)) and received less frequent (57.9% vs. 75%) mineralocorticoid receptor antagonists (MRAs) as compared to those in Group 2 (eGFR ≥ 60 mL/min/1.73 m^2^). RDW demonstrated better prognostic value in predicting combined mortality and rehospitalization outcomes in Group 2 patients (area under the curve: 0.70; 95% CI (0.62–0.80)) compared to those in Group 1 (area under the curve: 0.53; 95% CI (0.35–0.72)). No statistically significant differences (*p* = 0.579) were observed in survival between patients with high (≥15%) and low (<15%) RDW values in the overall population, though trends favored worse outcomes with elevated RDW. Similarly, no significant differences (*p* = 0.374) were observed in survival between patients with high (Group 2) and low (Group 1) eGFR values. **Conclusions:** RDW appears to be a meaningful prognostic biomarker for HF patients, particularly in those without CKD. Further multicenter studies are needed to validate its clinical utility and potential for guiding treatment in this high-risk population.

## 1. Introduction

Chronic kidney disease (CKD) has an important role in the development and management of heart failure (HF) due to the interdependent relationship between kidney and heart function. First of all, HF and CKD share common risk factors such as hypertension, diabetes mellitus, smoking, and obesity. Secondly, in patients with CKD, the impaired ability of the kidneys to regulate salt and water balance leads to fluid retention, which exacerbates HF by increasing venous congestion and afterload on the heart [1]. This can result in systemic and pulmonary edema, further impairing cardiac output. Additionally, CKD often triggers neurohormonal mechanisms, such as overactivation of the renin–angiotensin–aldosterone system (RAAS) and the sympathetic nervous system (SNS), which promotes hypertension, fibrosis, and adverse cardiac remodeling [2]. The prevalence of HF increases as kidney function declines, with nearly 45% of patients undergoing dialysis also experiencing HF, half of whom have reduced ejection fraction [3]. Not surprisingly, in the majority of CKD patients, the risk of developing CVD outweighs the hazard of progression to advanced or end-stage kidney disease. The overlapping pathophysiological mechanisms between CKD and HF, including inflammation, oxidative stress, and neurohormonal dysregulation, create a vicious cycle that complicates treatment and worsens prognosis [2]. Therefore, clinical research must be encouraged in patients with combined renal and cardiac diseases to accomplish evidence-based management and improve their outcomes.

Red blood cell distribution width (RDW) is a measure of the variability in the size of circulating red blood cells (RBCs) routinely included in a complete blood count (CBC) and is typically used to classify several types of anemia [4]. In recent years, however, RDW has emerged as a significant marker in cardiovascular diseases, particularly in HF [5], since an elevated value has been associated with several pathophysiological mechanisms, including inflammation, oxidative stress, neurohormonal activation, and impaired erythropoiesis [6]. Importantly, higher RDW values have been linked to worse outcomes in HF patients [7]. Studies have demonstrated that RDW is an independent prognostic marker of mortality [8,9,10,11], rehospitalization [10,12], and disease severity [13] in both acute and chronic HF. RDW’s prognostic value extends across the HF spectrum, including patients with reduced (HFrEF) and preserved ejection fraction (HFpEF) [14]. However, there are still debates about its utility and prognostic value in HF, particularly between patients with CKD and patients with normal renal function, as the former group of patients (CKD patients) are traditionally excluded from HF studies [2].

The aim of this original research was to investigate the prognostic value of RDW on all-cause mortality and HF rehospitalizations in patients with HF and evaluate its association with patients’ baseline eGFR.

## 2. Methods

### 2.1. Study Design

The present work was an observational retrospective study including patients hospitalized for acute decompensation of HF at a tertiary university hospital (University Hospital of Larissa, Greece) (April 2022–March 2023). It was conducted in accordance with the Declaration of Helsinki and approved by the institutional review board (IRB) of the University of Thessaly (protocol code: 53326; date of approval: 29 November 2023). Informed consent was waived due to the retrospective nature of the study.

### 2.2. Patients and Setting

The study included patients using the following criteria: i. HF; ii. age >18 years; and iii. admitted to the hospital for dyspnea on minimal exertion or rest dyspnea with an oxygen saturation <90% on admission in arterial blood gas with one or more of the following: (a) signs of congestion (third heart sound or pulmonary rales >1/3 or lower extremity/sacral edema >1+ on examination); (b) interstitial congestion or pleural effusion on chest X-ray; and (c) increased natriuretic peptides.

Exclusion criteria were as follows: i. systolic blood pressure <90 mmHg; ii. severe valvular disease; iii. complex congenital heart disease; iv. active cancer; v. sepsis; vi. estimated glomerular filtration rate (eGFR) < 20 mL/min/1.73 m^2^; and vii. missing data.

All patients were separated into either Group 1 (eGFR < 60) or Group 2 (eGFR ≥ 60) depending on their initial renal function during hospital admission. All patients were treated for acute decompensation of HF.

Blood samples were taken to determine renal function indices and other biochemical markers upon emergency department admission and during follow-up. Levels of hemoglobin (Hgb) and RDW were measured with the use of a Unicel DxH 900 Hematology Analyzer (Beckman, Indianapolis, IN, USA) on samples obtained for an evaluation of the standard of care (upper limit of normal was 15% in our laboratory), while urea, creatinine, and electrolytes (i.e., sodium and potassium) were measured with the use of a Cobas 8000 (Roche, Mannheim, Germany). The eGFR was calculated based on the CKD-EPI Creatinine Equation (2021) [15]. The transthoracic echocardiography examinations were evaluated by two independent echocardiographers with the use of a Vivid T8 v206 (General Electric Medical Systems, Wuxi, China). The left ventricular ejection fraction (EF) was calculated with the biplane “methods of discs” according to the current guidelines of the European Association of Cardiovascular Imaging [16]. Comorbidities (coronary artery disease, hypertension, atrial fibrillation, and diabetes mellitus) and medications (neurohormonal inhibitors and diuretics) were derived from patients’ medical records.

### 2.3. Endpoints

The primary endpoint was the combined all-cause mortality and HF rehospitalizations during the follow-up, while secondary endpoints were all-cause mortality and HF rehospitalizations, separately.

### 2.4. Statistical Analysis

Baseline characteristics were assessed for normality using the D’ Agostino and Pearson test of normal distribution. Comparisons were made using the unpaired *t*-test for parametric data and the Mann–Whitney test for nonparametric data, respectively, regarding the continuous outcomes. Categorical outcomes were compared using the chi-square test.

We evaluated the discrimination traits of RDW for mortality, rehospitalization, and combined outcomes in the total population in patients with a low (<60 mL/min/1.73 m^2^) and high (≥60 mL/min/1.73 m^2^) eGFR. Discrimination was estimated by constructing receiver operating characteristic (ROC) curves and by calculating the area under the ROC curve (AUC). The AUC was considered by calculating the 95% confidence intervals [17]. We defined poor, fair, and excellent model discrimination traits as the AUC levels of <0.70, 0.70–0.79, and 0.80–1.00, respectively [17].

We also compared the event-free survival for all-cause death and HF rehospitalization as well as the overall survival between patients with high (≥15%) and low (<15%) RDW levels and a high (≥60 mL/min/1.73 m^2^) and low (<60 mL/min/1.73 m^2^) eGFR by constructing a Kaplan–Meier graph over the follow-up period. The log-rank test was used for the comparison of the survival distributions between the 2 groups. A *p*-value < 0.05 was set as the threshold indicating a statistically important result.

All data were analyzed using Microsoft^®^ Excel 16.61 (Microsoft, Redmond, Washington, DC, USA), Prism^®^ Graphpad 10.0.3 for Mac (GraphPad Software, San Diego, CA, USA), and SPSS statistical software (version 26.0, Armonk, NY, USA: IBM Corp).

## 3. Results

The study included 171 patients in total, with median age of 79 (69–85) years, median urea of 51 (38.5–79) mg/dL, and median creatinine of 1.1 (0.9–1.6) mg/dL. The mean follow-up period was 6.1 months (SD = 4.8 months).

Patients were separated into two groups according to a cut-off value of their eGFR (60 mL/min/1.73 m^2^); the first group with the worse renal function (eGFR < 60 mL/min/1.73 m^2^) consisted of 95 patients with a median age of 80 (73–86) years, median urea of 72 (57–101) mg/dL, and median creatinine of 1.5 (1.2–2.0) mg/dL, while the second group with the better renal function (eGFR ≥ 60 mL/min/1.73 m^2^) consisted of 76 patients with a median age of 75 (62–83) years, median urea of 39 (32–45) mg/dL, and median creatinine of 0.8 (0.7–1.0) mg/dL. The most frequent comorbidity in the total population was arterial hypertension (HTN), followed by atrial fibrillation (AF), coronary artery disease (CAD), and diabetes mellitus (DM). The majority of patients received b-blockers, mineralocorticoid receptor antagonists (MRAs), and loop diuretics (furosemide). Sodium–glucose cotransporter-2 inhibitors (SGLT2is) and angiotensin-converting enzyme inhibitors (ACE-is)/angiotensin receptor blockers (ARBs) were prescribed in a lower number of patients while sacubitril/valsartan was the least prescribed drug among the others. Patients in Group 1 (eGFR < 60 mL/min/1.73 m^2^) were older, manifested higher median RDW values, and received less frequent mineralocorticoid receptor antagonists (MRAs) as compared to those in Group 2 (eGFR ≥ 60 mL/min/1.73 m^2^). Table 1 demonstrates baseline characteristics of the total sample and baseline characteristics within each group.

**Figure 1:**
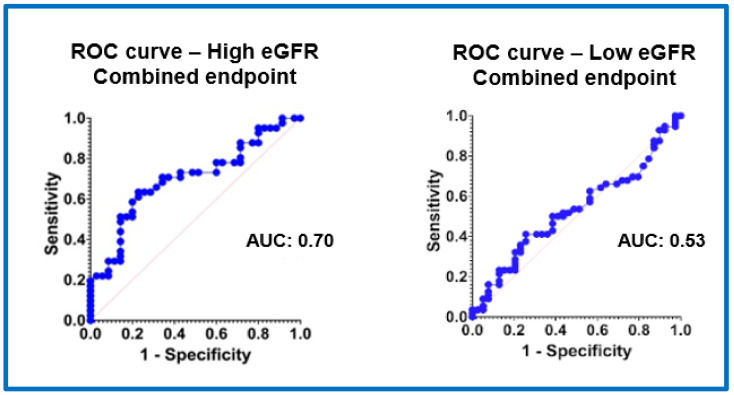
Discrimination traits of RDW for the combined primary outcome (all-cause mortality and HF rehospitalization) in patients with a high eGFR (≥60 mL/min/1.73 m^2^) and low eGFR (<60 mL/min/1.73 m^2^), estimated by constructing receiver operating characteristic (ROC) curves and by calculating the area under the ROC curve (AUC). Poor, fair, and excellent model discrimination traits are defined as AUC levels of <0.70, 0.70–0.79, and 0.80–1.00, respectively.

**Figure 2:**
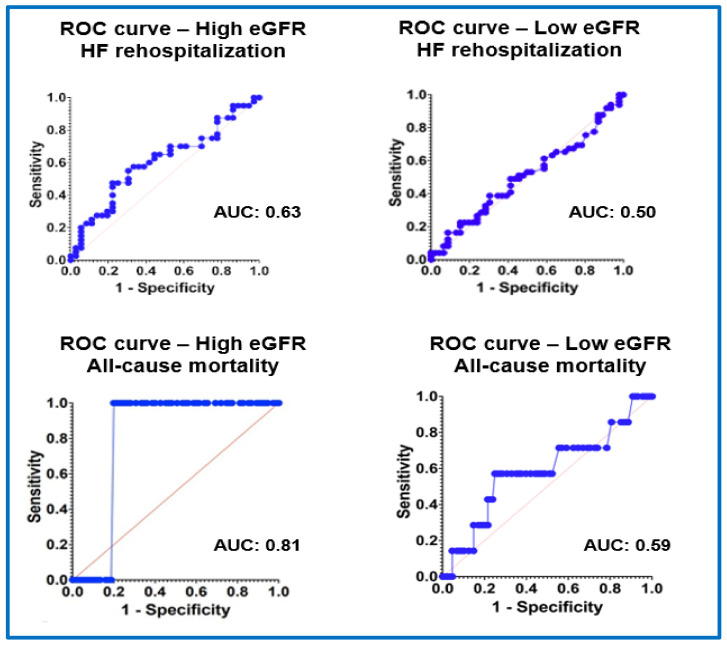
Discrimination traits of RDW for the components of the primary endpoint (HF rehospitalization and all-cause mortality) in patients with a high eGFR (≥60 mL/min/1.73 m^2^) and low eGFR (<60 mL/min/1.73 m^2^), estimated by constructing receiver operating characteristic (ROC) curves and by calculating the area under the ROC curve (AUC).

Discrimination traits of RDW for the primary combined outcome, HF rehospitalization, and all-cause mortality are demonstrated in detail in Figure 1 and Figure 2 and Supplementary Materials Figure S1. RDW demonstrated better prognostic value in predicting combined mortality and rehospitalization outcomes in Group 2 patients (area under the curve: 0.70; 95% CI (0.62–0.80)) compared to those in Group 1 (area under the curve: 0.53; 95% CI (0.35–0.72)).

Event-free survival regarding all-cause mortality and HF rehospitalization between patients with a high (≥60 mL/min/1.73 m^2^) and low (<60 mL/min/1.73 m^2^) eGFR and RDW levels (≥15% vs. <15%) were compared using a Kaplan–Meier graph (Supplementary Materials Figures S2 and S3). No statistical significance was observed (high vs. low eGFR *p* = 0.268; high vs. low RDW levels *p* = 0.818).

**Figure 3:**
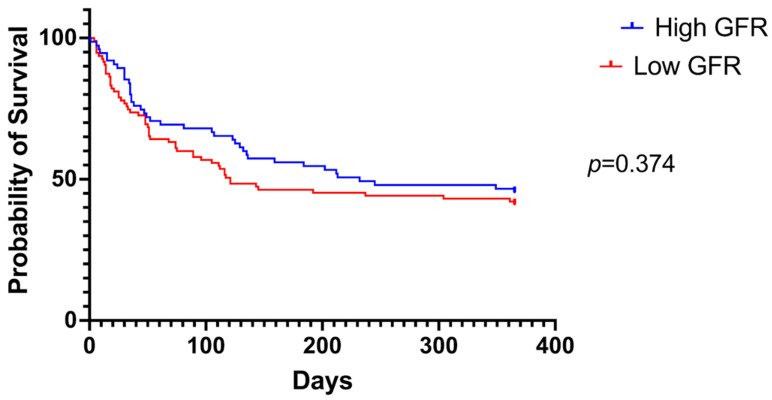
A comparison of overall survival between patients with higher and lower eGFR levels using a Kaplan–Meier graph.

**Figure 4:**
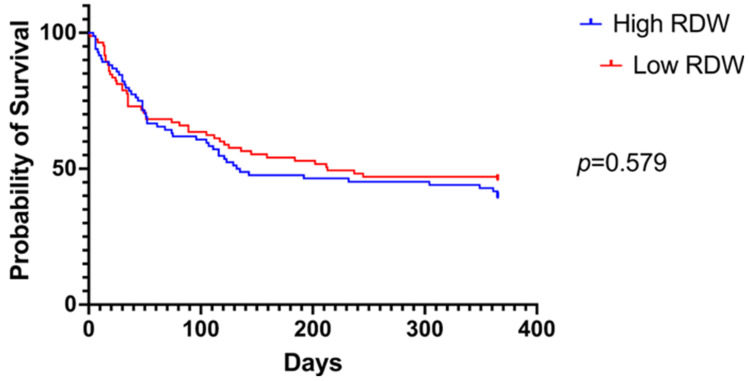
A comparison of overall survival between patients with higher and lower RDW levels using a Kaplan–Meier graph.

Overall survival between patients with higher and lower eGFR and RDW levels were also compared using a Kaplan–Meier graph (Figure 3 and Figure 4, respectively). Although survival seemed to be better in patients with a higher eGFR and lower RDW, no statistical significance between the two groups was observed in each case (high vs. low eGFR *p* = 0.374; high vs. low RDW levels *p* = 0.579).

## 4. Discussion

The present observational clinical study investigated the prognostic value of RDW on combined all-cause mortality and HF rehospitalizations, as well as in all-cause mortality and HF rehospitalizations separately in patients with HF, and evaluated its association with patients’ eGFR. Our study demonstrated that RDW, a prognostic biomarker in HF, seems to have better prognostic value in patients with HF and normal renal function (eGFR ≥ 60 mL/min/1.73 m^2^) compared to HF patients with renal failure (eGFR < 60 mL/min/1.73 m^2^).

Patients with a high severity of HF usually present high in-hospital mortality, increased likelihood of readmission, and increased mortality after discharge [18,19]. Moreover, due to the fact that they are treated with high doses of diuretics as a first-line therapy in HF, patients may present CKD stages 3 and 4, nephrotic syndrome with an impaired glomerular filtration rate (GFR), or even end-stage CKD [20,21]. Similarly, patients with CKD frequently suffer from HF, and the management of HF in these patients may be challenging because HF medications may further impair renal function [22,23]. CKD is defined by measuring the estimated GFR (eGFR), urinalysis, and albuminuria quantification [24]. In our study, we separated patients according to an eGFR cut-off point of 60 mL/min/1.73 m^2^ because this value is indicative of CKD, even when evidence of kidney damage such as albuminuria is absent [2]. The prevalence of HF increases as kidney function declines, with nearly 50% of CKD stage 4 and 5 patients suffering from CVD [25]. A decrease in the eGFR in CKD patients is associated with a higher risk for new onset HF, occurring in approximately 17–21% of these patients [26], while the prognosis of CKD patients with HF is also poor and worsens with deteriorating renal function [27]. From a pathophysiological view, HF and CKD usually co-exist, as the CKD-induced systemic inflammation leads to impaired vascular endothelial function, hypertension, atherosclerosis, vascular calcification, and myocardial fibrosis [28]. Similarly, hemodynamic processes in HF, such as decreased cardiac output and increased systemic venous pressure, further increase salt and fluid retention, augment systemic and renal congestion, and cause renal dysfunction [29]. Moreover, neurohormonal changes in HF, including RAAS and SNS overactivity, and CVD-related processes lead to further progression of CKD and aggravation of inflammatory processes [30,31]. Oxidative stress, systematic inflammation, and endoplasmic reticulum stress are all mechanisms contributing to cardiac and renal fibrosis [32]. Lastly, both CKD and HF are frequently associated with anemia due to erythropoietin impairment, uremia-induced inhibitors of erythropoiesis, reduced erythrocyte survival, and pathologic iron homeostasis [33], resulting in neurohormonal overactivity and thus further deterioration of renal and cardiac function [34].

RDW is a measure of the variability in the size of circulating RBCs used to classify types of anemia, with higher RDW values indicating greater variation in RBC size, a condition known as anisocytosis [4,35]. RDW can be calculated either as the standard deviation (RDW-SD) or as the coefficient of variation (RDW-CV) of erythrocyte volumes, with normal values ranging between 11.5 and 15% [5,36]. Except for hematological diseases, RDW has also been used as a significant prognostic marker in cardiovascular diseases, especially in HF in recent years [5]. RDW is associated with indices of cardiac function including the natriuretic peptides [37], peak oxygen consumption [38], and LV echocardiographic indices [39,40]. RDW is also associated with clinical outcomes in HF. The first large prospective study which associated RDW and HF was performed by Felker et al. [7], who measured RDW levels in 2679 chronic HF patients and found that each increase in SD by one in RDW was related to a 17% higher risk of cardiovascular mortality or HF hospitalization [hazard ratio (HR), 1.17; 95% confidence interval (95% CI), 1.10–1.25] and a 12% higher risk (HR = 1.12; 95% CI: 1.03–1.20) of all-cause death. One year later, Tonelli et al. [41] presented similar results, as they found that each 1% rise in the RDW value related to a 14% increase in all-cause mortality (HR = 1.14; 95% CI: 1.05–1.24) and a 15% increase in the risk (HR = 1.15; 95% CI: 1.05–1.26) of developing symptomatic HF on follow-up. In the following years, large meta-analyses came to confirm all these findings, with Huang et al. showing a 10% higher risk of future mortality events (HR = 1.10; 95% CI: 1.07–1.13) [9]; Shao et al. showing a 19% higher risk of major adverse cardiovascular events (HR = 1.19; 95% CI: 1.08–1.30), a 12% higher risk of mortality (HR = 1.12; 95% CI: 1.08–1.16), and a 9% higher risk of hospitalization (HR = 1.09, 95% CI: 1.03–1.16) [10]; and Hou et al. showing an 11% higher risk of mortality (HR = 1.11; 95% CI: 1.04–1.14) and an 11% higher risk of HF in patients with pre-existing cardiovascular disease (HR = 1.11; 95% CI: 1.05–1.17) [42] for each 1% increase in the RDW value, respectively. Many other prospective and retrospective studies over the years found a significant prognostic value of serial assessment of RDW over time in HF endpoints, including mortality and rehospitalizations, that may be more clinically meaningful and informative than the admission value of RDW [8,43,44,45,46,47,48]. Interestingly, a recent trial from our institute demonstrated that each 1% increase in the RDW value at admission was independently associated with worse prognosis both in non-diabetic (HR = 1.14; 95% CI: 1.01–1.29) and diabetic (1.35; 95% CI: 1.12–1.62) patients who were admitted to the emergency department for acute HF, while the longitudinal changes in RDW revealed a significant interaction with diabetes (β coefficient, −0.002; *p* = 0.042), showing that metabolic imbalances may have an impact on longitudinal changes in RDW [13]. Finally, RDW levels are shown to have a U-shaped connection with 30-day mortality, and they are linked to an elevated risk of short-, medium-, and long-term all-cause mortality among chronic HF patients [11], while increased RDW values are also significantly associated with a higher risk of 3-month readmission in hospitalized patients with HF [12].

As far as pathophysiology is concerned, anisocytosis may have a pivotal role in the onset and exacerbation of HF. The erythrocyte size heterogeneity may represent a severe dysfunction of this essential corpuscular blood element. In cases of high anisocytosis, RBCs are frequently presented with lower deformability and impaired oxygen-carrier capacity, thus contributing to the reduced oxygenation of cardiomyocytes, whilst abnormal erythrocytes may also contribute to the development of cardiac fibrosis through the promotion or amplification of inflammation, cardiomyocyte stress, and apoptosis [35]. Patients with HF and a low eGFR tend to experience higher levels of inflammation, oxidative stress, and impaired erythropoiesis, which are all associated with increased RDW [49,50,51]. These pathophysiological mechanisms are more pronounced in CKD, potentially impairing the prognostic value of RDW in this subgroup. Inflammation, in combination with neurohormonal system activation, may disturb the red blood cell membrane, therefore changing RBC maturation and leading to increased RDW [6]. Indeed, studies have reported an association between RDW and inflammatory indices, including interleukin-6 (IL-6) and C-reactive protein (CRP) [52,53].

Inflammation and oxidative stress play a key role in CKD. The reactive oxygen species (ROS) which are produced during oxidative stress in CKD may damage the RBC membrane, leading to reduced cell deformability and an increased destruction of RBCs [54,55]. There are more mechanisms promoting anisocytosis and elevated RDW levels in patients with renal disease. Erythropoietin and iron deficiency in CKD are among them. Most specifically, erythropoietin, which is a hormone critical for stimulating RBC production in the bone marrow, is produced insufficiently by the kidneys in advanced CKD, resulting in a diminished and irregular production of RBCs, smaller RBCs, and thus an increased RDW [56]. Similarly, iron deficiency in CKD leads to impaired hemoglobin synthesis, the production of both microcytic and hypochromic RBCs, and an increased RDW [57]. Moreover, hepcidin, a liver-derived hormone that regulates iron absorption and release, is often elevated in renal disease due to inflammation, reducing iron availability, further impairing erythropoiesis, and contributing to anisocytosis [58]. Other proposed mechanisms of an RDW increase in CKD include vitamin deficiencies [59], uremic toxins [60], dialysis-related hemolysis [61], and hypoxia-induced immature RBC production [62]. Specifically, vitamin deficiency in CKD due to poor nutritional status, including deficiencies in key nutrients required for RBC production, such as iron, folic acid, and vitamin B12, may cause microcytic and/or macrocytic anemia that significantly increases RDW [59]. Hemodialysis in advanced CKD patients can cause hemolysis, leading to an increased release of immature RBCs, while the inflammatory response triggered by dialysis may further impair erythropoiesis and contribute to anisocytosis [61]. Finally, reduced oxygen delivery due to anemia and impaired erythropoiesis stimulates the release of immature RBCs from the bone marrow in an attempt to compensate for reduced oxygen-carrying capacity, contributing to increased RDW. Taking all the above mechanisms into consideration, it seems that RDW may serve as a robust biomarker for predicting mortality and rehospitalizations in HF patients without kidney dysfunction [36,49]. Our findings came to confirm this hypothesis by showing that RDW tends to have prognostic value in patients with HF and a high eGFR for clinical outcomes such as all-cause mortality and HF hospitalizations.

There are strengths and limitations in our study. Firstly, it was an observational retrospective study without any interventions and, as a result, no safe conclusions could be extracted in these subpopulations. Secondly, data on the albumin levels in the urine were available only in a minority (≈10%) of patients and therefore, a more comprehensive stratification of kidney impairment was not feasible. Similarly, potential confounders such as hepcidin, erythropoietin (EPO), or fibroblast growth factor 23 (FGF-23) were not available. The fact that our sample size was small makes our results non-generalizable. Notably, the present analysis was “hypothesis generating”, as it was not powered to demonstrate statistical significance in survival differences for RDW levels. However, it highlighted the importance of RDW in HF and its potential to be used as a prognostic marker in HF patients with and without CKD utilizing “real world data”. This finding could provide significant insights in the field of HF.

In summary, the present study demonstrates the excellent performance of RDW as a prognostic marker in patients with HF and a higher eGFR (i.e., ≥60 mL/min/1.73 m^2^), which is not observed in HF patients with lower eGFR values (i.e., <60 mL/min/1.73 m^2^). Therefore, RDW should be used with caution as a prognostic tool in the latter group of patients (i.e., those with HF and CKD).

## 5. Conclusions

RDW, a prognostic biomarker in HF for clinical outcomes such as all-cause mortality and rehospitalizations, demonstrated in the present observational work better prognostic value in patients with HF and preserved renal function compared to HF patients with a low eGFR. Nevertheless, future multicenter clinical trials with a larger number of patients are required in order to extract safe conclusions.

## Figures and Tables

**Table 1:** Baseline characteristics of the total sample and baseline characteristics within each group.

Baseline Characteristics	Total Population	Group 1: eGFR < 60	Group 2: eGFR ≥ 60	*p*-Value
Number of patients	171	95	76	
Females (%)	49.1	53.7	43.4	0.18
Age, years	79 (69–85)	80 (73–86)	75 (62–83)	0.006
EF	35 (21.3–48.8)	35 (20.0–46.3)	35 (23.8–50.0)	0.978
NYHA III-IV (%)	90.6	89.5	92.1	0.56
HFrEF (%)	60.2	58.9	61.8	0.93
HFmrEF (%)	6.4	8.4	3.9	
HFpEF (%)	33.4	32.7	34.3	
Blood sample indices				
Urea (mg/dL)	51 (38.5–79)	72 (57–101)	39 (32–45)	<0.001
Creatinine (mg/dL)	1.1 (0.9–1.6)	1.5 (1.2–2.0)	0.8 (0.7–1.0)	<0.001
eGFR (mL/min/1.73 m^2^)	55.9 (36.7–76.0)	36.3 (30.4–46.2)	75.5 (64.4–88.3)	<0.001
Sodium (mmol/L)	138 (134–140)	138 (134–140)	138 (135–140)	0.81
Potassium (mmol/L)	4.5 (4.1–5.0)	4.5 (4.2–5.0)	4.4 (4.0–4.9)	0.11
Hemoglobin (g/dL)	11.9 (10.4–13.1)	11.6 (10.30–12.90)	12.1 (10.6–13.2)	0.18
Red blood cell distribution Width (%)	16.2 (14.6–18.4)	16.6 (15.0–18.8)	15.6 (14.1–17.8)	0.009
Comorbidities				
CAD (%)	48.5	53.7	42.1	0.17
HTN (%)	96.0	93.4	98.0	0.24
AF (%)	57.9	52.6	62.1	0.22
DM (%)	35.7	27.6	42.1	0.06
Medication				
SGLT2i (%)	32.1	29.5	35.5	0.42
ACE-i/ARB (%)	33.9	29.5	39.5	0.20
B-blockers (%)	89.5	90.5	88.2	0.63
MRA (%)	65.5	57.9	75.0	0.02
Furosemide (%)	84.2	87.4	80.3	0.21
Sacubitril/valsartan (%)	25.1	22.1	29.0	0.38

Abbreviation; eGFR, estimated glomerular filtration rate; CAD, coronary artery disease; HTN, arterial hypertension; AF, atrial fibrillation; DM, diabetes mellitus; SGLT2i, sodium–glucose transport protein 2 inhibitor; ACE-i, angiotensin-converting enzyme inhibitor; ARB, angiotensin receptor blocker; MRA, mineralocorticoid receptor antagonist.

## Data Availability

The original contributions presented in the study are included in the article/Supplementary Materials; further inquiries can be directed to the corresponding author.

## Supplementary Materials

The following supporting information can be downloaded at: https://www.mdpi.com/article/10.3390/jcm13237395/s1, Figure S1: Receiver operating characteristic (ROC) curves (a) for the combined endpoint (all-cause mortality and HF rehospitalization), (b) for HF rehospitalization, and (c) for all-cause mortality in the total population; Figure S2: Comparison of event-free survival (all-cause mortality and HF rehospitalization) between patients with higher and lower eGFR levels using a Kaplan–Meier graph; Figure S3: Comparison of event-free survival (all-cause mortality and HF rehospitalization) between patients with higher (≥15%) and lower (<15%) RDW levels using a Kaplan–Meier graph.
