# Supplementary material for: The Prognostic Role of RDW in Hospitalized Heart Failure Patients with and Without Chronic Kidney Disease
Source: J Clin Med. 2024 Dec 4;13(23):7395. doi: 10.3390/jcm13237395 (PMC11642004; doi:10.3390/jcm13237395)
Supplement: Supplementary file 1 [file jcm-13-07395-s001.zip › jcm-3347172-supplementary.pdf]

Supplementary Materials

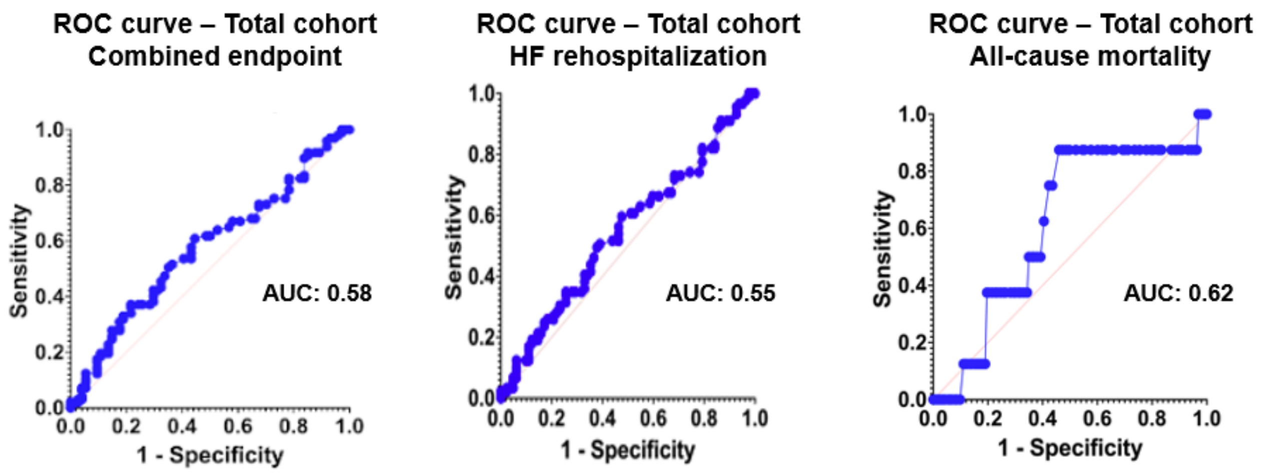

Figure S1: Receiver-operating characteristic (ROC) curves a) for the combined endpoint (all-cause mortality and HF rehospitalization), b) for HF rehospitalization, c) for all-cause mortality, in the total population

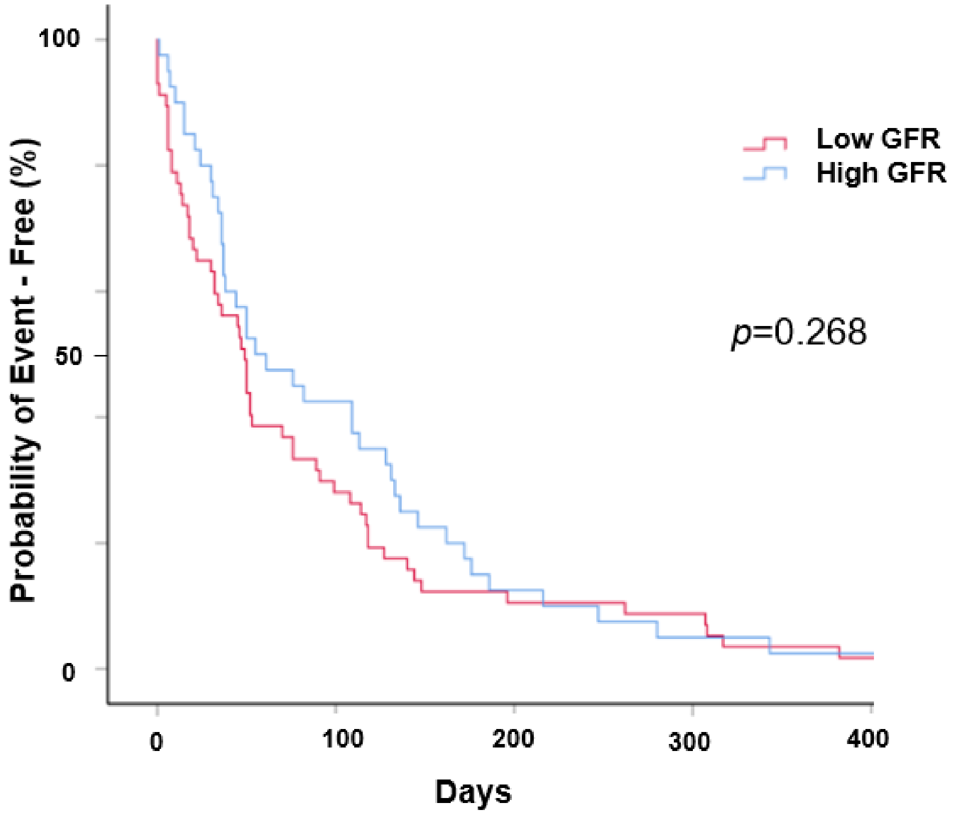

Figure S2: Comparison of event-free survival (all-cause mortality and HF rehospitalization) between patients with higher (≥60 mL/min/1.73m^2) and lower (<60 mL/min/1.73m^2) estimated GFR levels, using a Kaplan-Meier graph. GFR: Glomerular Filtration Rate

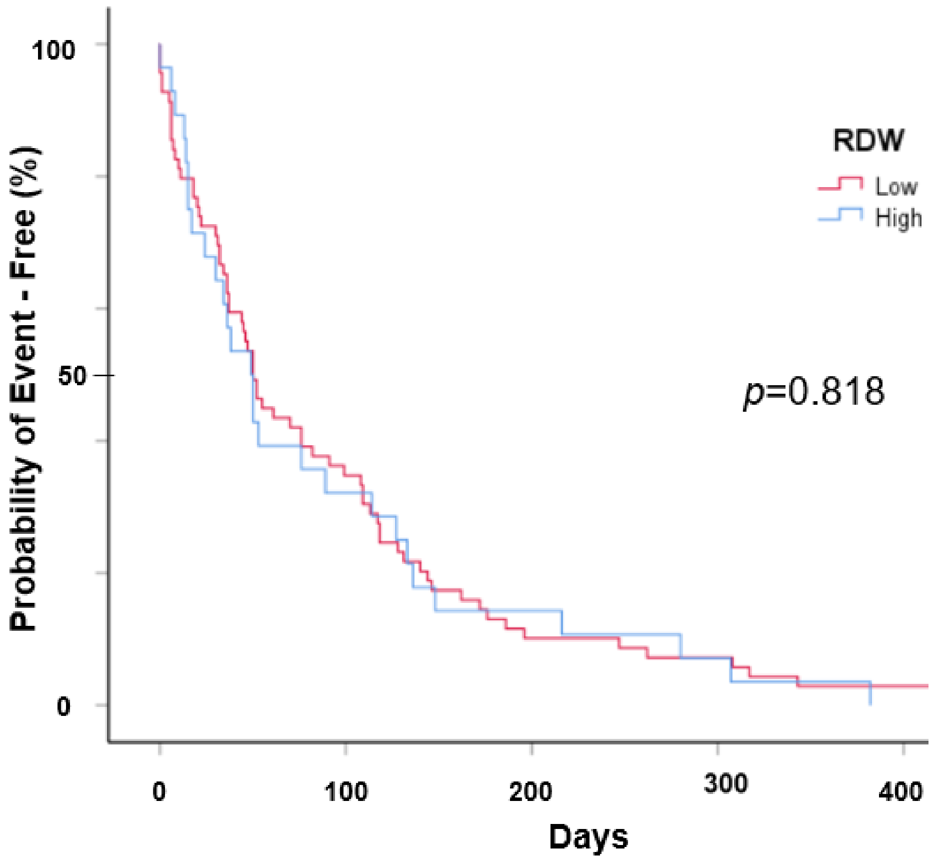

Figure S3. Comparison of event-free survival (all-cause mortality and HF rehospitalization) between patients with higher (≥15%) and lower (<15%) RDW levels, using a Kaplan-Meier graph. RDW: Red Blood cell Distribution Width
